# Long-term weight gain in obese COPD patients participating in a disease management program: a risk factor for reduced health-related quality of life

**DOI:** 10.1186/s12931-021-01787-9

**Published:** 2021-08-14

**Authors:** Manuel B. Huber, Nelli Schneider, Florian Kirsch, Larissa Schwarzkopf, Anja Schramm, Reiner Leidl

**Affiliations:** 1 Institute of Health Economics and Health Care Management, Helmholtz Zentrum München, Neuherberg, Germany; 2 Institute for Medical Information Processing, Biometry, and Epidemiology (IBE), Ludwig-Maximilians-University, Munich, Germany; 3 Munich School of Management and Munich Center of Health Sciences, Ludwig-Maximilians-Universität, Munich, Germany; 4 Comprehensive Pneumology Center Munich (CPC-M), Member of the German Center for Lung Research (DZL), Neuherberg, Germany; 5 IFT – Institute fuer Therapieforschung, Working Group Therapy and Health Services Research, Leopoldstrasse 175, 80804 Munich, Germany; 6 AOK Bayern, Service Center of Health Care Management, Regensburg, Germany

**Keywords:** COPD, BMI, Health-related quality of life, Obesity, Longitudinal study, Real-world evidence

## Abstract

**Background:**

Little is known about how long-term weight gain affects the health perception of COPD patients.

**Objectives:**

The aim is to evaluate the long-term association of BMI change and health-related quality of life (HRQoL) in obese COPD patients.

**Methods:**

Claims and survey data from a COPD disease management program were used to match two groups of COPD patients with BMI ≥ 30 who have differing weight trajectories over a 5-year timespan via propensity score and genetic matching. EQ-5D-5L, including visual analog scale (VAS) and COPD Assessment Test (CAT), were used as outcomes of interest. Sociodemographic and disease-based variables were matched.

**Results:**

Out of 1202 obese COPD patients, 126 with a weight increase of four or more BMI points were matched separately with 252 (propensity score matching) and 197 (genetic matching) control subjects who had relatively stable BMI. For the EQ-5D-5L, patients with BMI increase reported significantly worse health perception for VAS and all descriptive dimensions except pain/discomfort. For the CAT, especially the perception of ability to complete daily activities and overall energy results were significantly worse. VAS differences reach the range of minimal important differences. Stopping smoking and already being in obesity class II were the most influential risk factors for BMI increase.

**Conclusion:**

Obese COPD patients who gain four or more BMI points over 5 years report significantly lower results in different dimensions of generic and disease-specific HRQoL than their peers with stable BMI. To improve real-world outcomes, tracking and preventing specific BMI trajectories could constitute a clinically relevant aspect of managing COPD patients.

**Supplementary Information:**

The online version contains supplementary material available at 10.1186/s12931-021-01787-9.

## Background

Chronic obstructive pulmonary disease (COPD) is a complex disease that is primarily characterized by airflow limitation but also systemic manifestations that lead to comorbidities [1]. COPD results from inflammation or alterations in repair mechanisms of the lung and can initiate or worsen comorbid diseases [1, 2]. This complicates patient management and affects patient outcomes by lowering health-related quality of life (HRQoL) and increasing mortality [3].

There is evidence that COPD is associated with nutritional and metabolic abnormalities and, in addition, poor nutrition is considered as a risk factor for COPD [4]. Simultaneously, the prevalence of obesity as well as overweight is increasing in developed and developing countries [5]. Furthermore, among over 400 prognostic COPD models for hard clinical outcomes, BMI is the fourth most frequently used predictor following age, FEV_1_, and sex [6]. In cross-sectional perspective, high BMI is associated with reduced HRQoL in COPD [7, 8]; regarding development over time, evidence is rare and not specific [9], indicating a research gap.

Improving HRQoL is an explicit goal in German disease management guidelines [10]. Factors that predict current HRQoL in COPD include, but are not limited to, previous HRQoL results, physical activity, exacerbations, and specific symptoms [11, 12]. Preventing BMI increase or finding ways to normalize it may therefore represent a key management aspect that is treated insufficiently in several COPD disease guidelines [13, 14] or is covered rather casually [15]. Taken together, BMI gain represents a fundamental aspect of disease management in COPD that has the potential to improve patient health perception, when specific criteria are met. Going beyond a previous study that evaluated the effect of absolute BMI values on health perception in COPD in a cross-sectional setting [8], the aim of this study is to evaluate the association between BMI development and HRQoL in a longitudinal context.

## Methods

### Data

This study uses claims data from AOK Bayern, a statutory health insurance fund, that were linked to survey data. Data were retrieved for all patients who participated in the COPD disease management program (DMP), a structured treatment/surveillance program for COPD patients. More information about the data can be found elsewhere [8]. Overall, the survey was sent to 49,664 DMP patients, 14,753 (29.7%) of whom responded. Observations with missing HRQoL or unrealistic values were removed. As underweight and obesity are very different entities that require different management approaches, especially in COPD, only patients with BMI ≥ 30 were included in this study. Following this filtering, 1202 patients remained for evaluation. The ethics committee of the Ludwig Maximilians University, Munich, approved the study (vote no. 17-358). Participants provided written informed consent at the time of inclusion in the DMP.

### Disease severity

Disease severity was assessed via the GOLD COPD staging system [16]. GOLD stages are based on the percentage of predicted forced FEV_1_ and require one post-bronchodilator diagnosis of COPD. The four GOLD severity stages are defined as 1 = mild (> 80% of FEV_1_ predicted), 2 = moderate (50–79% predicted), 3 = severe (30–49% predicted), and 4 = very severe (< 30% predicted).

### Measures

BMI is expressed in units of kg/m^2^. HRQoL quantifies health states as a key indicator of patient-reported outcome, and can be used to improve clinical care [17]. BMI classes I, II, and III are defined as BMI ranging from ≥ 30 to < 35, ≥ 35 to < 40, and ≥ 40 respectively. It is recommended to use both generic as well as disease-specific instruments to evaluate the influence of COPD on HRQoL [18]. The EQ-5D-5L was used to describe and evaluate generic HRQoL [19]. The descriptive part is directed at specific aspects of health and includes the dimensions of mobility, self-care, usual activity, pain/discomfort, and anxiety/depression. In the 5L version of the EQ-5D, respondents are asked to report one out of five problem levels in each dimension ranging from no problems to extreme problems. For overall evaluation, the patient’s response to the visual analog scale (VAS) is taken as part of the EQ-5D-5L, thus reflecting general health perception. As a continuous scale, the VAS ranges from 0 for the worst imaginable health state to 100 for the best. The use of EQ-5D-5L VAS in COPD patients has been validated psychometrically [19]. The COPD Assessment Test (CAT) was used to measure disease-specific HRQoL [20]. The eight dimensions of the CAT refer, among others, to cough, mucus production, sleep, and energy. Each dimension has six answer levels from best (0) to worst (5). Overall, the CAT sum score ranges between 0 for the best health and 40 for the worst.

### Model

To evaluate the association of BMI change over time, we used two different matching approaches: propensity score matching (PSM) [21] and genetic matching (GM) [22]. The latter combines PSM and Mahalanobis distance matching and serves to improve the robustness of study results. Standardized mean differences and respective p-values were calculated for all pairwise comparisons. Control subjects—those with a relatively stable BMI—are defined as having a BMI change in the range of ± 1 points from year five prior to the survey (t0) to the year of the survey (t1). Case patients are defined as having a BMI increase of at least four points from t0 to t1. HRQoL values are from t1, whereas BMI trajectory is based on the fifth year before as well as the last year before the survey. The time span of 5 years was selected because it was also incorporated in a comparable evaluation for weight development in COPD, based on the scientifically well-established Copenhagen City Heart Study [23]. Furthermore, this constellation provided the highest number of observations. To improve precision [24] and because of the large number of available control subjects, the matching ratio was set to 2:1 for control (stable BMI) and case (BMI increase of 4 or more points) patients. The terms “stable BMI” and “BMI increase” will refer to each respective group in this paper and will not be used in a different context. The variables education and HRQoL are evaluated from survey data, whereas the variables age (years), sex, smoking status (active, ex, non-smoker during DMP), sleep apnea and, depending on their availability, severe exacerbations that required a hospital visit, and medium exacerbations that required a doctor’s visit, and all single diseases of the Charlson Comorbidity index 5 years prior to the survey [25] are evaluated from claims data. All variables are used to match both groups at t0. The number of quit attempts was not accounted for in the models, but the change in smoking status was controlled for. Physicians documented when patients switched from being active smokers to being ex-smokers in the DMP. To identify risk factors for the two weight trajectories, we calculated a logistic regression model with binary BMI group as dependent outcome. McFadden’s pseudo R-squared was calculated as a performance measure for this logistic regression model. McFadden’s pseudo R-squared values between 0.2 and 0.4 are considered to be a very good fit [26]. Moreover, to improve robustness of the results and to account for events that significantly influence HRQoL in the short term, we also ran a linear regression model that controlled for the number of hospital visits that were coded based on the following ICD-10 chapters [27] in the year before the survey: ‘Chapter I: Certain infectious and parasitic diseases’, ‘Chapter III: Diseases of the blood and blood-forming organs and certain disorders involving the immune mechanism’, ‘Chapter VI: Diseases of the nervous system’, ‘Chapter VII: Diseases of the eye and adnexa’, ‘Chapter XII: Diseases of the skin and subcutaneous tissue’, ‘Chapter XIV: Diseases of the genitourinary system’, ‘Chapter XVIII: Symptoms, signs and abnormal clinical and laboratory findings, not elsewhere classified’, ‘Chapter XIX: Injury, poisoning and certain other consequences of external causes’, and ‘Chapter XXI: Factors influencing health status and contact with health services’.

To compare group means for illustration purposes, we used independent parametric t-tests and examine the required assumptions. We used the Shapiro–Wilk test to control for normality and F-tests to examine homogeneity in variances. All analyses were performed in the software environment R [28].

## Results

A total of 1202 patients were available for the evaluation: 1076 had stable BMI and 126 had BMI increase. Following the matching procedure, based on standardized mean difference (SMD), significant differences in matched variables between both groups disappeared (Table 1). However, significant differences between both groups existed for several unmatched variables, including generic and disease-specific HRQoL. VAS and all descriptive dimensions of the EQ-5D-5L except pain/discomfort differed, while half of the CAT dimensions [4–6, 8], especially those affecting activity and energy levels, differed significantly as well. There was no significant difference in central indictors of disease severity such as FEV_1_ predicted or number of severe exacerbations.

**Table 1:** Sociodemographic and disease-related data of study participants pre- and post-matching

	Pre-matching			Post-matching PSM			Post-matching GM		
	Stable BMI	BMI increase	p	Stable BMI	BMI increase	p	Stable BMI	BMI increase	p
n	1076	126		252	126		197	126	
Age (SD)	70.65 (8.85)	63.65 (9.27)	< 0.001	64.27 (8.78)	63.65 (9.27)	0.524	65.49 (9.10)	63.65 (9.27)	0.079
Male (%)	683 (63.5)	73 (57.9)	0.263	156 (61.9)	73 (57.9)	0.527	125 (63.5)	73 (57.9)	0.381
*Education (%)*			0.981			0.885			0.480
High school	890 (82.7)	104 (82.5)		201 (79.8)	104 (82.5)		167 (84.8)	104 (82.5)	
Middle school	89 (8.3)	12 (9.5)		28 (11.1)	12 (9.5)		20 (10.2)	12 (9.5)	
Academic high school	18 (1.7)	2 (1.6)		6 (2.4)	2 (1.6)		3 (1.5)	2 (1.6)	
University	17 (1.6)	2 (1.6)		7 (2.8)	2 (1.6)		0 (0.0)	2 (1.6)	
Other	62 (5.8)	6 (4.8)		10 (4.0)	6 (4.8)		7 (3.6)	6 (4.8)	
*GOLD stage (%)*			0.243			0.882			0.843
GOLD stage 1	150 (13.9)	25 (19.8)		46 (18.3)	25 (19.8)		32 (16.2)	25 (19.8)	
GOLD stage 2	580 (53.9)	62 (49.2)		134 (53.2)	62 (49.2)		103 (52.3)	62 (49.2)	
GOLD stage 3	300 (27.9)	36 (28.6)		65 (25.8)	36 (28.6)		56 (28.4)	36 (28.6)	
GOLD stage 4	46 (4.3)	3 (2.4)		7 (2.8)	3 (2.4)		6 (3.0)	3 (2.4)	
BMI (SD)	34.10 (3.73)	36.25 (5.55)	< 0.001	35.79 (4.71)	36.25 (5.55)	0.407	35.03 (4.21)	36.25 (5.55)	0.027
Active smokers (%)	234 (21.7)	55 (43.7)	< 0.001	109 (43.3)	55 (43.7)	1.000	71 (36.0)	55 (43.7)	0.211
*Total no. of exacerbations (5 years before)* Severe exacerbations (SD)	0.05 (0.24)	0.08 (0.30)	0.171	0.07 (0.30)	0.08 (0.30)	0.713	0.08 (0.30)	0.08 (0.30)	0.925
Medium exacerbations (SD)	0.60 (1.57)	0.51 (1.13)	0.505	0.46 (1.33)	0.51 (1.13)	0.752	0.42 (1.07)	0.51 (1.13)	0.464
Sleep apnea (%)	123 (11.4)	22 (17.5)	0.069	40 (15.9)	22 (17.5)	0.806	29 (14.7)	22 (17.5)	0.616
*Comorbidities (5 years before)* Myocardial infarction	45 (4.2)	3 (2.4)	0.461	8 (3.2)	3 (2.4)	0.914	5 (2.5)	3 (2.4)	1.000
Congestive heart failure	251 (23.3)	34 (27.0)	0.422	66 (26.2)	34 (27.0)	0.967	51 (25.9)	34 (27.0)	0.929
Peripheral vascular disease	197 (18.3)	22 (17.5)	0.911	46 (18.3)	22 (17.5)	0.962	28 (14.2)	22 (17.5)	0.529
Cerebrovascular accident	162 (15.1)	9 (7.1)	0.023	19 (7.5)	9 (7.1)	1.000	16 (8.1)	9 (7.1)	0.914
Rheumatic disease (%)	60 (5.6)	8 (6.3)	0.880	14 (5.6)	8 (6.3)	0.938	9 (4.6)	8 (6.3)	0.657
Peptic ulcer disease	19 (1.8)	3 (2.4)	0.892	6 (2.4)	3 (2.4)	1.000	4 (2.0)	3 (2.4)	1.000
Mild liver disease	266 (24.7)	25 (19.8)	0.271	52 (20.6)	25 (19.8)	0.964	43 (21.8)	25 (19.8)	0.774
Diabetes mellitus (uncomplicated)	413 (38.4)	52 (41.3)	0.594	115 (45.6)	52 (41.3)	0.487	70 (35.5)	52 (41.3)	0.358
Diabetes mellitus (complicated)	202 (18.8)	23 (18.3)	0.983	45 (17.9)	23 (18.3)	1.000	29 (14.7)	23 (18.3)	0.492
Renal disease	137 (12.7)	17 (13.5)	0.920	34 (13.5)	17 (13.5)	1.000	27 (13.7)	17 (13.5)	1.000
Cancer	95 (8.8)	8 (6.3)	0.440	19 (7.5)	8 (6.3)	0.832	10 (5.1)	8 (6.3)	0.812
VAS	55.06 (19.76)	49.46 (20.64)	**0.003**	54.64 (19.97)	49.46 (20.64)	**0.019**	56.37 (20.10)	49.46 (20.64)	**0.003**
*EQ-5D-5L* Mobility (SD)	2.59 (1.09)	2.81 (1.11)	**0.032**	2.53 (1.09)	2.81 (1.11)	**0.019**	2.43 (1.07)	2.81 (1.11)	**0.002**
Self-care (SD)	1.76 (1.02)	2.00 (1.19)	**0.013**	1.69 (1.00)	2.00 (1.19)	**0.009**	1.64 (0.89)	2.00 (1.19)	**0.002**
Usual activities (SD)	2.42 (1.11)	2.70 (1.13)	**0.009**	2.40 (1.13)	2.70 (1.13)	**0.018**	2.34 (1.06)	2.70 (1.13)	**0.004**
Pain/discomfort (SD)	2.75 (0.99)	2.88 (1.09)	0.180	2.81 (1.00)	2.88 (1.09)	0.549	2.67 (0.96)	2.88 (1.09)	0.069
Anxiety/depression (SD)	1.93 (1.05)	2.27 (1.12)	**0.001**	1.98 (1.07)	2.27 (1.12)	**0.014**	1.97 (1.07)	2.27 (1.12)	**0.016**
*CAT* Cough	2.65 (1.16)	2.76 (1.17)	0.298	2.56 (1.12)	2.76 (1.17)	0.097	2.64 (1.14)	2.76 (1.17)	0.372
Phlegm	2.67 (1.24)	2.61 (1.26)	0.640	2.64 (1.23)	2.61 (1.26)	0.837	2.73 (1.21)	2.61 (1.26)	0.394
Chest tightness	2.17 (1.30)	2.22 (1.32)	0.644	2.11 (1.33)	2.22 (1.32)	0.426	2.17 (1.26)	2.22 (1.32)	0.735
Breathlessness	3.75 (1.17)	3.98 (1.12)	**0.029**	3.73 (1.18)	3.98 (1.12)	**0.046**	3.72 (1.16)	3.98 (1.12)	0.047
Home activities	2.60 (1.39)	2.91 (1.35)	**0.015**	2.50 (1.42)	2.91 (1.35)	**0.008**	2.48 (1.35)	2.91 (1.35)	**0.005**
Confidence leaving home	1.62 (1.39)	1.92 (1.48)	**0.024**	1.49 (1.34)	1.92 (1.48)	**0.005**	1.49 (1.31)	1.92 (1.48)	**0.006**
Soundness of sleep	2.52 (1.37)	2.71 (1.52)	0.154	2.47 (1.40)	2.71 (1.52)	0.131	2.44 (1.33)	2.71 (1.52)	0.099
Energy level	2.88 (1.14)	3.15 (1.27)	**0.011**	2.85 (1.15)	3.15 (1.27)	**0.021**	2.91 (1.10)	3.15 (1.27)	0.065
CAT score (SD)	20.84 (7.55)	22.27 (8.04)	**0.046**	20.35 (7.62)	22.27 (8.04)	**0.024**	20.59 (7.30)	22.27 (8.04)	0.052
Severe exacerbation year before (SD)	0.10 (0.49)	0.09 (0.36)	0.786	0.11 (0.52)	0.09 (0.36)	0.646	0.05 (0.25)	0.09 (0.36)	0.223
Exacerbations over 4 years (SD)	0.26 (1.03)	0.28 (0.93)	0.886	0.22 (0.81)	0.28 (0.93)	0.550	0.16 (0.66)	0.28 (0.93)	0.194
FEV_1_% predicted (SD)	57.46 (22.07)	57.05 (20.31)	0.843	60.96 (24.08)	57.05 (20.31)	0.118	59.09 (23.09)	57.05 (20.31)	0.418

Significance level of 5 % are in bold

*SD* standard deviation, *VAS* visual analogue scale, *CAT* COPD Assessment Test, *FEV*_*1*_ Forced expiratory volume in 1 s

Additional file 1: Figure S1 depicts VAS results for both BMI groups, stratified by GOLD stages, as well as the respective significance levels for the implemented t-tests to compare group means. Mean VAS results were not significantly different between both BMI trajectories in all GOLD stages.

Table 2 depicts results of the logistic regression model with binary BMI trajectory as dependent outcome. McFadden’s R-squared for this model is 0.174. Patients aged 60 years and older had significantly lower odds ratios (ORs) for increasing BMI, and the respective risks decreased by age group. Although class II obesity was associated with an OR of 1.6, ex-smokers had the highest OR with 2.1.

**Table 2:** Logistic regression results, binary BMI trajectory as dependent outcome

Variable	Estimate	SE	p-value	OR	2.5%	97.5%
(Intercept)	− 0.187	0.592	0.752	0.829	0.253	2.643
*Age (years)* Age 50–60	− 0.743	0.576	0.197	0.476	0.155	1.521
Age 60–70	− 1.312	0.575	**0.022**	0.269	0.088	0.859
Age 70–80	− 1.959	0.600	**0.001**	0.141	0.044	0.471
Age > 80	− 4.163	1.173	**0.000**	0.016	0.001	0.114
*BMI group* Class II obesity	0.477	0.238	**0.045**	1.612	1.005	2.559
Class III obesity	0.575	0.310	0.064	1.777	0.952	3.222
Male	− 0.261	0.223	0.243	0.770	0.498	1.197
*Highest education level* Secondary school	− 0.387	0.367	0.292	0.679	0.315	1.344
Academic high school	0.011	0.785	0.989	1.011	0.153	3.895
University	− 0.129	0.896	0.885	0.879	0.105	3.970
Other education levels	− 0.389	0.484	0.421	0.677	0.238	1.631
*GOLD stage (5 years before)* GOLD stage 2	− 0.322	0.285	0.259	0.725	0.419	1.286
GOLD stage 3	− 0.008	0.324	0.981	0.992	0.528	1.889
GOLD stage 4	− 0.707	0.688	0.304	0.493	0.105	1.681
*Smoking status*						
Never smoker during DMP	− 0.745	0.246	**0.002**	0.475	0.294	0.773
Ex-smoker	0.750	0.362	**0.038**	2.117	1.028	4.273
Active smoker	0.505	0.540	0.350	1.657	0.536	4.594
*Total no. of exacerbations (5 years before)* Severe exacerbations	0.563	0.370	0.129	1.756	0.808	3.536
Medium exacerbations	− 0.111	0.075	0.142	0.895	0.762	1.027
Sleep apnea	0.423	0.287	0.140	1.527	0.855	2.639
*Charlson index (5 years before)* Myocardial infarction	− 0.842	0.641	0.189	0.431	0.098	1.314
Congestive heart failure	0.437	0.246	0.076	1.548	0.946	2.492
Peripheral vascular disease	0.319	0.293	0.277	1.376	0.761	2.414
Cerebrovascular accident	− 0.537	0.389	0.168	0.584	0.256	1.195
Rheumatic disease (%)	0.373	0.440	0.397	1.452	0.573	3.283
Peptic ulcer disease	0.441	0.682	0.518	1.554	0.334	5.265
Mild liver disease	− 0.434	0.259	0.093	0.648	0.383	1.059
Diabetes mellitus (uncomplicated)	0.285	0.251	0.255	1.330	0.807	2.162
Diabetes mellitus (complicated)	− 0.198	0.316	0.531	0.820	0.436	1.513
Renal disease	0.526	0.318	0.098	1.692	0.884	3.093
Cancer	− 0.093	0.430	0.829	0.911	0.361	1.993

Significance level of 5 % are in bold

*SE* standard error, *OR* odds ratio, *2.5%* and *97.5%* confidence intervals in the last two columns

Table 3 depicts the results of the multivariate linear regression model with VAS as dependent outcome. The adjusted R-squared for this model is 0.098. Old age, other education levels, class III obesity, active smoking, sleep apnea, having “symptoms, signs, and abnormal clinical and laboratory findings, not elsewhere classified”, and BMI increase were all associated with significant decline in VAS results. Being male and having “diseases of the blood and blood-forming organs and certain disorders involving the immune mechanism” were associated with higher VAS results.

**Table 3:** Multivariate linear regression results, VAS as dependent outcome, no matching

Variable	Estimate	Std. error	Statistic	p-value
(Intercept)	63.286	5.816	10.882	0.000
*Age (years)* Age 50–60	− 5.180	4.903	− 1.057	0.291
Age 60–70	− 4.998	4.813	− 1.039	0.299
Age 70–80	− 8.099	4.870	− 1.663	0.097
Age > 80	− 14.204	5.153	− 2.756	**0.006**
Male	3.975	1.228	3.236	**0.001**
*Highest education level* Secondary school	− 2.180	2.041	− 1.068	0.286
Academic high school	0.551	4.332	0.127	0.899
University	− 2.660	4.434	− 0.600	0.549
Other education levels	− 6.579	2.477	− 2.656	0.**008**
*GOLD stage (5 years before)* GOLD stage 2	0.391	1.790	0.218	0.827
GOLD stage 3	− 3.943	2.230	− 1.768	0.077
GOLD stage 4	− 4.226	3.619	− 1.168	0.243
*BMI group* Class II obesity	− 0.986	1.367	− 0.721	0.471
Class III obesity	− 4.888	2.016	− 2.425	**0.015**
*Smoking status (5 years before)* Active during DMP	− 3.375	1.390	− 2.427	**0.015**
*Total no. of exacerbations (5 years before)* Severe exacerbations	− 2.847	2.404	− 1.184	0.237
Medium exacerbations	0.036	0.376	0.097	0.923
Sleep apnea	− 4.344	1.786	− 2.432	0.015
*Charlson Index (5 years before)* Myocardial infarction	− 2.907	2.920	− 0.996	0.320
Congestive heart failure	− 0.774	1.394	− 0.556	0.579
Peripheral vascular disease	0.402	1.591	0.253	0.800
Cerebrovascular accident	− 3.280	1.677	− 1.956	0.051
Rheumatic disease (%)	− 1.716	2.504	− 0.685	0.493
Peptic ulcer disease	− 1.336	4.111	− 0.325	0.745
Mild liver disease	− 0.567	1.329	− 0.427	0.670
Diabetes mellitus (uncomplicated)	0.660	1.398	0.472	0.637
Diabetes mellitus (complicated)	− 0.500	1.756	− 0.285	0.776
Renal disease	− 2.778	1.784	− 1.557	0.120
Cancer	− 1.100	2.021	− 0.544	0.586
*ICD-10 diagnoses (1 year before)*				
Chapter I: certain infectious and parasitic diseases	0.415	2.705	0.153	0.878
Chapter III: diseases of the blood (…)	6.776	2.472	2.741	**0.006**
Chapter V: mental and behavioral disorders	− 2.988	1.935	− 1.544	0.123
Chapter VI: diseases of the nervous system	− 0.921	1.312	− 0.702	0.483
Chapter VII: diseases of the eye and adnexa	− 4.972	3.903	− 1.274	0.203
Chapter XII: diseases of the skin and subcutaneous tissue	− 3.976	2.109	− 1.885	0.060
Chapter XIV: diseases of the genitourinary system	1.757	1.076	1.633	0.103
Chapter XVIII: symptoms (…)	− 4.599	1.271	− 3.619	**0.000**
Chapter XIX: injury, poisoning (…)	− 1.911	1.839	− 1.039	0.299
Chapter XXI: factors influencing health status	− 0.158	0.992	− 0.159	0.874
Severe exacerbations (1 year before)	− 0.266	1.447	− 0.184	0.854
FEV_1_% predicted	0.054	0.033	1.645	0.100
BMI increase	− 5.473	1.890	− 2.895	**0.004**

Significance level of 5 % are in bold

Diseases of the blood (…): Diseases of the blood and blood-forming organs and certain disorders involving the immune mechanism; Symptoms (…): Symptoms, signs and abnormal clinical and laboratory findings, not elsewhere classified; Injuries (…): Injury, poisoning and certain other consequences of external causes

## Discussion

Of 1202 obese COPD patients in a DMP, this study found that 10.48% increased their BMI by four or more points over a 5-year period. Using two different matching approaches and implementing a linear regression model to predict VAS results, this BMI increase is shown to be associated with lower generic and disease-specific health perception. Comparing our findings with results from previous literature is difficult as, to our knowledge, this is the first study to evaluate the association between BMI trajectories and HRQoL in COPD. Although solid literature is available for the association between cross-sectional BMI values and HRQoL [7, 29, 30], literature evaluating the association between BMI change and HRQoL in COPD is rare. An evaluation based on the Copenhagen City Heart Study concludes that weight gain > 3 BMI points in obese COPD patients is associated with increased mortality, but only in patients with severe COPD and not the rest [23]. However, mortality and HRQoL are two different entities that can hardly be compared.

## Clinical implications

To be clinically relevant, a change in health perception should reach the threshold of so-called minimum important differences (MIDs). The MID for the EQ-5D-5L VAS was found to be 6.9 (6.5–8.0) points in COPD [19]. In the context of this study, this means that VAS differences between stable BMI and BMI increase patients reach the range of MIDs. On average, COPD patients with stable BMI have 5- to 7-point higher VAS results than their control subjects with BMI increase. This falls into the range of the respective VAS MID in COPD and is therefore most probably clinically relevant. There was no significant difference between GOLD stage, VAS, and the two BMI trajectories (Additional file 1: Fig. S1), indicating that the loss of HRQoL is observable irrespective of COPD severity.

Effective prevention of weight gain in at-risk patients could offer a management option that prevents significant deterioration in HRQoL and should therefore be accounted for. Beyond COPD, it has been found difficult to improve health outcomes once obesity has already been reached or further increased [31]. This underlines the relevance of preventing weight gain, which is linked to worse health outcome. To identify patients who are at risk of BMI increase, we implemented a logistic regression model on the binary outcome “BMI trajectory” (Table 2). We clearly see that obesity grade II and being an ex-smoker are significant risk factors for BMI increase, whereas older age or being a never-smoker decrease the respective risk. Hence, COPD patients who have already gained too much weight and who stop smoking are prone to continue on this trajectory. Detecting these patients in clinical practice is important and can be achieved by monitoring weight change and smoking status over time. This can be done in person, while suitable software could also be used to warn physicians when COPD patients are at risk.

Further weight gain in already obese people has also been observed to reduce generic HRQoL in the general population, as a large Australian study showed for the physical component summary of the SF-36 [32]. Yet, in our study, obese COPD patients with weight gain were not only found to report a decline in generic HRQoL, but also in the lung-specific dimensions measured by the CAT.

## Strengths and limitations

The strengths of this study include the novelty of the research question as well as the dataset, which consists of claims as well as survey data. Moreover, this is one of the first studies, if not the first study, to evaluate the association between weight gain and HRQoL in COPD patients over time. As weight gain can be prevented and generic as well as disease-specific HRQoL is negatively associated, this represents an important management aspect of COPD that is currently only partly accounted for in COPD guidelines.

Patients who enrolled in the COPD DMP were observed in two time periods 5 years apart, which results from the study design. On the basis of our study, we cannot assume similar effects for a shorter or longer observation period. Additional research is needed in this regard. Our analysis is also subject to several limitations. Participation in the AOK COPD DMP was voluntary, and therefore self-selection effects are likely. However, as patients with better health consciousness are more likely to participate in a DMP [33], the problems observed in this cohort could be more present as well as more relevant in the non-DMP population. We only modeled the association of two BMI trajectories on the HRQoL of COPD patients and therefore may miss associations for other trajectories. However, we added a sensitivity analysis to the supplement section (Additional file 1: Table S1) to account for a second trajectory (BMI increase ≥ 3) that led to comparable results. Although we controlled for many relevant variables, we cannot fully exclude factors that reduce HRQoL but are independent of BMI change. However, we tried to account for all possible relevant events in the year before the survey.

In absence of t0 results for HRQL we could not include corresponding information in our matching process. Hence the observed results more rather reflect associations than a causal relationship between “weight gain and HRQL decline”. Furthermore a potential reverse causation, meaning that already reduced HRQL might have induced weight gain cannot be fully ruled out. Reverse causation, in which already reduced HRQoL may drive weight gain, could not be controlled for as HRQoL results at t0 are not available and surveys were not provided at this point in time. HRQoL results at t0 would allow us to account for possible differences in VAS results at the beginning of the evaluation period. However, to partly address this issue, we thoroughly matched the populations and accounted for a wide variety of variables at t0 that are associated with HRQoL loss, for example comorbidities, smoking status, COPD severity, and exacerbations, to name a few. T0 results for HRQoL were not available. To reduce the probability of reverse causation, we matched the populations across several relevant variables that influence health perception. Our results point toward an association between weight gain and reduced HRQoL, but causal relationships cannot be proven based on the existing study design.

The BMI itself does not provide information about the body composition of the observed individuals. A higher BMI within the observation period of 5 years could be caused by gaining weight in the form of both fat-free mass and muscle mass [23]. However, it is unlikely that older patients and those in the medium to severe stages of COPD gain lots of fat-free muscle mass. In our observation group, only obese COPD patients with a BMI ≥ 30 are included. Therefore, the results are only representative of already obese patients. However, COPD patients who gain four or more BMI points are at risk of significantly reduced HRQoL. Hence, these findings represent a notable management aspect as physicians can take preventive action to stabilize or reduce the weight of COPD patients during DMP participation.

## Conclusion

For obese COPD patients, our study shows that weight gain of four or more BMI points over a 5-year period is significantly associated with lower generic and disease-specific HRQoL. Obesity class II and stopping smoking are risk factors significantly associated with BMI increase.

Preventing BMI increase in obese COPD patients could therefore constitute an important management option to avoid significant deterioration in HRQoL.

## Supplementary Information

**Additional file 1: Table S1.** Sociodemographic and disease-related data of study participants pre- and post-matching, BMI increased defined as BMI change ≥ 3 points. **Figure S1.** Boxplots of VAS results post-matching PSM, stratification by GOLD stage, t-test for group means, BMI ≥ 30

## Data Availability

The datasets generated and/or analyzed during the current study are not publicly available according to the data protection concept approved by the responsible data security officials and the ethics committee.
