# Supplementary material for: Long-term weight gain in obese COPD patients participating in a disease management program: a risk factor for reduced health-related quality of life
Source: Respir Res. 2021 Aug 14;22:226. doi: 10.1186/s12931-021-01787-9 (PMC8364095; doi:10.1186/s12931-021-01787-9)

Additional

| **Table S1** Sociodemographic and disease-related data of study participants pre- and post-matching,  BMI increased defined as BMI change ≥ 3 points | | | | | | | | | | |
| --- | --- | --- | --- | --- | --- | --- | --- | --- | --- | --- |
|  | **Pre-matching** | | | **Post-matching PSM** | | | **Post-matching GM** | | | |
|  | Stable BMI | BMI increase | p | Stable BMI | BMI increase | p | Stable BMI | BMI increase | p | |
| n | 1,076 | 213 |  | 426 | 213 |  | 299 | 213 |  | |
| Age (years, SD) | 70.65 (8.85) | 64.37 (9.54) | **<0.001** | 65.78 (8.83) | 64.37 (9.54) | 0.065 | 66.29 (9.14) | 64.37 (9.54) | **0.022** | |
| Male (%) | 683 (63.5) | 119 (55.9) | **0.044** | 253 (59.4) | 119 (55.9) | 0.444 | 181 (60.5) | 119 (55.9) | 0.334 | |
| Education (%) |  |  | 0.722 |  |  | 0.991 |  |  | 0.986 | |
| High school | 890 (82.7) | 169 (79.3) |  | 345 (81.0) | 169 (79.3) |  | 242 (80.9) | 169 (79.3) |  | |
| Middle school | 89 (8.3) | 23 (10.8) |  | 43 (10.1) | 23 (10.8) |  | 31 (10.4) | 23 (10.8) |  | |
| Academic high school | 18 (1.7) | 5 (2.3) |  | 9 (2.1) | 5 (2.3) |  | 7 (2.3) | 5 (2.3) |  | |
| University | 17 (1.6) | 3 (1.4) |  | 6 (1.4) | 3 (1.4) |  | 3 (1.0) | 3 (1.4) |  | |
| Other | 62 (5.8) | 13 (6.1) |  | 23 (5.4) | 13 (6.1) |  | 16 (5.4) | 13 (6.1) |  | |
| GOLD stage (%) |  |  | 0.149 |  |  | 0.781 |  |  | 0.782 | |
| 1 | 150 (13.9) | 42 (19.7) |  | 72 (16.9) | 42 (19.7) |  | 52 (17.4) | 42 (19.7) |  | |
| 2 | 580 (53.9) | 109 (51.2) |  | 230 (54.0) | 109 (51.2) |  | 163 (54.5) | 109 (51.2) |  | |
| 3 | 300 (27.9) | 56 (26.3) |  | 109 (25.6) | 56 (26.3) |  | 73 (24.4) | 56 (26.3) |  | |
| 4 | 46 (4.3) | 6 (2.8) |  | 15 (3.5) | 6 (2.8) |  | 11 (3.7) | 6 (2.8) |  | |
| BMI (SD) | 34.10 (3.73) | 36.00 (5.21) | **<0.001** | 35.38 (4.48) | 36.00 (5.21) | 0.117 | 34.94 (4.15) | 36.00 (5.21) | **0.010** | |
| Active smokers (%) | 234 (21.7) | 81 (38.0) | **<0.001** | 146 (34.3) | 81 (38.0) | 0.397 | 89 (29.8) | 81 (38.0) | 0.063 | |
| Severe exacerbation (SD) | 0.05 (0.24) | 0.06 (0.26) | 0.457 | 0.05 (0.26) | 0.06 (0.26) | 0.744 | 0.02 (0.16) | 0.06 (0.26) | **0.029** | |
| Medium exacerbations (SD) | 0.60 (1.57) | 0.58 (1.27) | 0.848 | 0.53 (1.51) | 0.58 (1.27) | 0.640 | 0.37 (1.07) | 0.58 (1.27) | **0.040** | |
| Sleep apnea (%) | 123 (11.4) | 37 (17.4) | **0.022** | 70 (16.4) | 37 (17.4) | 0.851 | 43 (14.4) | 37 (17.4) | 0.427 | |
| Myocardial infarction | 45 (4.2) | 4 (1.9) | 0.158 | 8 (1.9) | 4 (1.9) | 1.000 | 6 (2.0) | 4 (1.9) | 1.000 | |
| Congestive heart failure | 251 (23.3) | 56 (26.3) | 0.401 | 117 (27.5) | 56 (26.3) | 0.826 | 61 (20.4) | 56 (26.3) | 0.145 | |
| Peripheral vascular disease | 197 (18.3) | 38 (17.8) | 0.949 | 83 (19.5) | 38 (17.8) | 0.695 | 46 (15.4) | 38 (17.8) | 0.536 | |
| Cerebrovascular accident | 162 (15.1) | 18 (8.5) | **0.015** | 35 (8.2) | 18 (8.5) | 1.000 | 24 (8.0) | 18 (8.5) | 0.993 | |
| Rheumatic disease (%) | 60 (5.6) | 10 (4.7) | 0.724 | 15 (3.5) | 10 (4.7) | 0.614 | 4 (1.3) | 10 (4.7) | **0.043** | |
| Peptic ulcer disease | 19 (1.8) | 3 (1.4) | 0.938 | 4 (0.9) | 3 (1.4) | 0.893 | 2 (0.7) | 3 (1.4) | 0.702 | |
| Mild liver disease | 266 (24.7) | 52 (24.4) | 0.993 | 105 (24.6) | 52 (24.4) | 1.000 | 77 (25.8) | 52 (24.4) | 0.810 | |
| Diabetes mellitus (uncomplicated) | 413 (38.4) | 84 (39.4) | 0.832 | 166 (39.0) | 84 (39.4) | 0.977 | 101 (33.8) | 84 (39.4) | 0.222 | |
| Diabetes mellitus (complicated) | 202 (18.8) | 34 (16.0) | 0.383 | 68 (16.0) | 34 (16.0) | 1.000 | 46 (15.4) | 34 (16.0) | 0.957 | |
| Renal disease | 137 (12.7) | 29 (13.6) | 0.811 | 64 (15.0) | 29 (13.6) | 0.721 | 41 (13.7) | 29 (13.6) | 1.000 | |
| Cancer | 95 (8.8) | 19 (8.9) | 1.000 | 36 (8.5) | 19 (8.9) | 0.960 | 18 (6.0) | 19 (8.9) | 0.282 | |
| VAS | 55.06 (19.76) | 52.06 (20.73) | **0.044** | 55.45 (20.09) | 52.06 (20.73) | **0.047** | 56.64 (20.87) | 52.06 (20.73) | | **0.014** |
| EQ_5DL_1 (SD) | 2.59 (1.09) | 2.75 (1.07) | 0.053 | 2.49 (1.07) | 2.75 (1.07) | **0.005** | 2.46 (1.10) | 2.75 (1.07) | **0.003** | |
| EQ_5DL_2 (SD) | 1.76 (1.02) | 1.90 (1.15) | 0.074 | 1.71 (0.98) | 1.90 (1.15) | **0.029** | 1.67 (0.98) | 1.90 (1.15) | **0.015** | |
| EQ_5DL_3 (SD) | 2.42 (1.11) | 2.63 (1.15) | **0.015** | 2.37 (1.08) | 2.63 (1.15) | **0.005** | 2.38 (1.08) | 2.63 (1.15) | **0.012** | |
| EQ_5DL_4 (SD) | 2.75 (0.99) | 2.90 (1.05) | 0.058 | 2.75 (1.00) | 2.90 (1.05) | 0.094 | 2.70 (1.01) | 2.90 (1.05) | **0.035** | |
| EQ_5DL_5 (SD) | 1.93 (1.05) | 2.17 (1.06) | **0.002** | 2.00 (1.11) | 2.17 (1.06) | 0.062 | 1.99 (1.08) | 2.17 (1.06) | 0.063 | |
| CAT1 | 2.65 (1.16) | 2.72 (1.13) | 0.387 | 2.59 (1.14) | 2.72 (1.13) | 0.162 | 2.62 (1.18) | 2.72 (1.13) | 0.340 | |
| CAT2 | 2.67 (1.24) | 2.69 (1.25) | 0.756 | 2.64 (1.23) | 2.69 (1.25) | 0.602 | 2.65 (1.23) | 2.69 (1.25) | 0.690 | |
| CAT3 | 2.17 (1.30) | 2.20 (1.32) | 0.746 | 2.17 (1.35) | 2.20 (1.32) | 0.786 | 2.10 (1.28) | 2.20 (1.32) | 0.397 | |
| CAT4 | 3.75 (1.17) | 3.94 (1.12) | **0.026** | 3.76 (1.15) | 3.94 (1.12) | 0.058 | 3.70 (1.16) | 3.94 (1.12) | **0.018** | |
| CAT5 | 2.60 (1.39) | 2.81 (1.40) | **0.043** | 2.53 (1.37) | 2.81 (1.40) | **0.018** | 2.50 (1.37) | 2.81 (1.40) | **0.014** | |
| CAT6 | 1.62 (1.39) | 1.86 (1.44) | **0.022** | 1.62 (1.36) | 1.86 (1.44) | **0.040** | 1.52 (1.35) | 1.86 (1.44) | **0.006** | |
| CAT7 | 2.52 (1.37) | 2.70 (1.48) | 0.085 | 2.51 (1.37) | 2.70 (1.48) | 0.117 | 2.37 (1.39) | 2.70 (1.48) | **0.010** | |
| CAT8 | 2.88 (1.14) | 3.09 (1.29) | **0.017** | 2.89 (1.15) | 3.09 (1.29) | **0.046** | 2.86 (1.16) | 3.09 (1.29) | **0.040** | |
| CAT score (SD) | 20.84 (7.55) | 22.01 (7.90) | 0.041 | 20.71 (7.58) | 22.01 (7.90) | 0.044 | 20.32 (7.64) | 22.01 (7.90) | 0.016 | |
| Severe exacerbation year before (SD) | 0.10 (0.49) | 0.08 (0.33) | 0.573 | 0.10 (0.42) | 0.08 (0.33) | 0.619 | 0.07 (0.42) | 0.08 (0.33) | 0.781 | |
| Exacerbations over 4 years (SD) | 0.26 (1.03) | 0.28 (0.96) | 0.865 | 0.22 (0.87) | 0.28 (0.96) | 0.457 | 0.19 (0.93) | 0.28 (0.96) | 0.308 | |
| FEV_1_ % predicted (SD) | 57.46 (22.07) | 58.46 (21.36) | 0.544 | 60.08 (23.16) | 58.46 (21.36) | 0.393 | 60.69 (22.84) | 58.46 (21.36) | 0.263 | |

**Figure S1** Boxplots of VAS results post-matching PSM, stratification by GOLD stage, t-test for group means, BMI ≥ 30


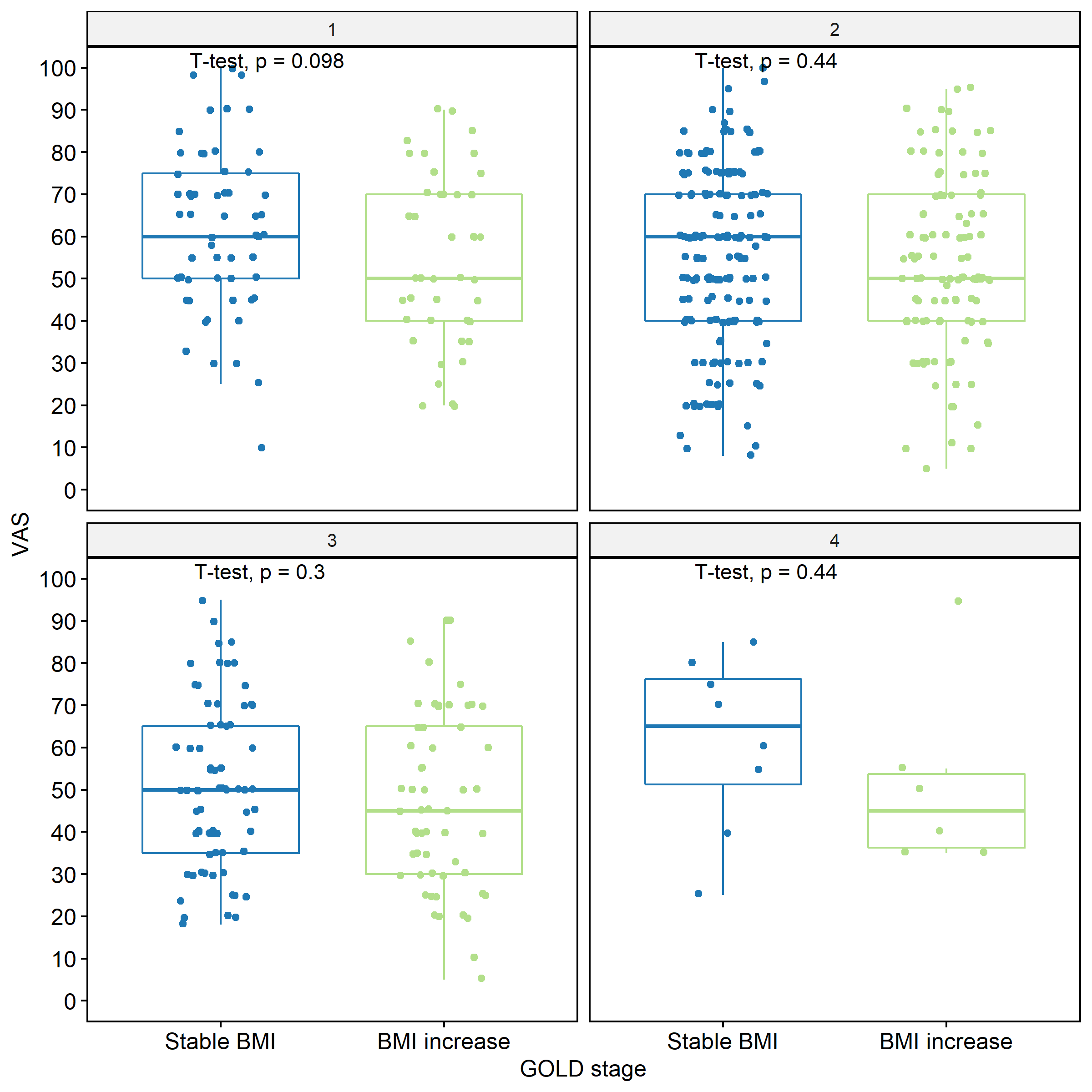

Supplement: Supplementary file 1 — Additional file 1: Table S1. Sociodemographic and disease-related data of study participants pre- and post-matching, BMI increased defined as BMI change ≥ 3 points. Figure S1. Boxplots of VAS results post-matching PSM, stratification by GOLD stage, t-test for group means, BMI ≥ 30 [file 12931_2021_1787_MOESM1_ESM.docx]
